# MG53, a Regenerative Myokine Linking Skeletal Muscle to Cardiac Repair

**DOI:** 10.3390/biom16040524

**Published:** 2026-04-01

**Authors:** Yuchen Chen, Kyung Eun Lee, Jongsoo Kim, Jae-Kyun Ko, Ki Ho Park

**Affiliations:** Division of Surgical Sciences, Department of Surgery, University of Virginia, Charlottesville, VA 22903, USA; kl2ev@virginia.edu (K.E.L.); qwe7xz@virginia.edu (J.K.); csk5aa@virginia.edu (J.-K.K.)

**Keywords:** MG53, TRIM72, membrane repair, muscle-derived myokine, inter-organ communication, cardioprotection, mitochondrial protection, regenerative medicine

## Abstract

Mitsugumin 53 (MG53, also TRIM72) is a muscle-enriched tripartite motif protein with a well-established role in acute membrane repair and cytoprotection in striated muscle and other stressed tissues. MG53 is a core component of cellular repair machinery, rapidly sensing membrane disruption and coordinating membrane resealing, mitochondrial preservation, and anti-inflammatory modulation. In contrast to its high expression in skeletal muscle, endogenous MG53 expression in the adult human heart is minimal, raising the question of how MG53 exerts cardioprotective effects in the human heart. Recent studies help address this by identifying MG53 as a circulating regenerative myokine. MG53 is secreted from skeletal muscle into the bloodstream and can reach distal organs, including the heart. These findings support a muscle-to-heart endocrine model in which MG53 mediates tissue crosstalk and helps provide repair capacity to the myocardium when intrinsic cardiac MG53 is low. Here, we summarize recent advances in MG53 biology, emphasizing molecular mechanisms and inter-organ communication underlying cardioprotection. We further highlight translational strategies leveraging recombinant MG53- and MG53-based therapeutics and discuss challenges that must be addressed for future clinical applications. Collectively, these insights support MG53 as an endocrine repair factor linking skeletal muscle to cardiac repair and a potential regenerative cardiovascular target.

## 1. Introduction

Skeletal muscle has emerged as a dynamic endocrine organ that communicates with distant tissues through the secretion of myokines [1,2,3,4], including irisin [5,6,7,8], myostatin [9,10], interleukin-6 (IL-6) [11,12,13,14], and Mitsugumin 53 (MG53, also TRIM72) [15,16,17]. These factors regulate systemic metabolism, immunity, and tissue homeostasis. MG53 is unique among these factors, functioning not only as a soluble myokine but also as a structural repair protein.

MG53 was first identified by Cai and colleagues in 2009 as a critical component of the plasma membrane repair machinery in striated muscle [18]. MG53-deficient mice exhibit defective membrane repair, muscle fragility, and impaired regenerative responses [18,19,20,21]. Subsequent studies revealed that MG53 is secreted into the circulation and detectable in serum at physiologically meaningful levels [22,23]. Further work demonstrated that elevated circulating MG53, either genetically or via recombinant protein, confers multiorgan cytoprotection [17,22,24,25,26,27,28,29,30,31,32,33,34,35,36,37,38,39,40,41,42,43,44,45,46,47,48,49,50,51,52,53,54,55,56,57,58,59], supporting the concept that MG53 functions not only as an intracellular repair protein but also as a regenerative myokine.

This endocrine role is especially important in the context of the human heart. The relative absence of MG53 in human cardiomyocytes raises important physiological and clinical considerations. Multiple transcriptomic and biochemical analyses show that MG53 expression in human myocardium is markedly lower than in murine hearts or human skeletal muscle [39,56,60]. This species-specific deficiency may contribute to the greater membrane fragility of the human heart under stress, potentially increasing its susceptibility to injury and its reliance on circulating MG53 for repair support. In this context, MG53 can be viewed as a unique myokine whose endocrine function provides a structural repair benefit to cardiac tissue, distinct from metabolic or immunomodulatory myokines traditionally implicated in cardiovascular physiology. In this review, we integrate current knowledge of MG53 biology by describing its structural organization and regulatory mechanisms, with particular attention to recent insights from cryo-electron microscopy studies, and by discussing the endocrine and myokine functions of circulating MG53 in tissue protection and repair, especially its unique role in human cardioprotection (Table 1). We further summarize emerging therapeutic strategies, including recombinant human MG53 (rhMG53), gene therapy, and biomaterial-based delivery, and highlight key unanswered questions and future directions for translating MG53 biology into therapeutic applications.

## 2. Structural and Molecular Biology of MG53

**Figure 1:**
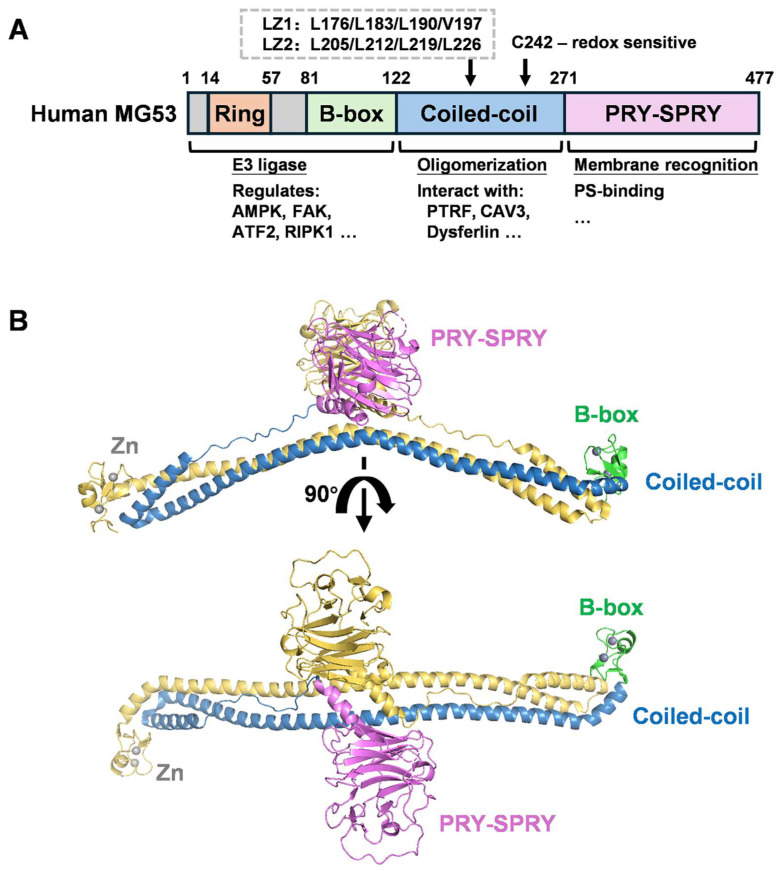
Overall structure of human MG53. (**A**) Schematic of MG53 domain organization. The RING, B-box, coiled-coil, and PRY-SPRY domains are colored in orange, green, blue, and pink, respectively. PS, phosphatidylserine. LZ, leucine-zipper motif. (**B**) Overall structure of MG53 homodimer (PDB: 7XT2) shown in two views. One monomer is colored yellow. Zn^2+^ is colored grey.

MG53 is a member of the tripartite motif (TRIM) protein family [18], characterized by an N-terminal RBCC module (RING finger, B-box, and coiled-coil region) followed by a C-terminal PRY-SPRY (SPla and the RYanodine receptor) domain [61,62,63] (Figure 1A). This modular architecture supports two core properties that define MG53 biology: (a) rapid assembly into repair-competent complexes at sites of membrane damage and (b) domain-dependent control of downstream signaling pathways linked to cellular stress responses. In this section, we summarize the structural features and domain-specific mechanisms that underlie MG53 function, focusing on how the RING domain supports ubiquitin-dependent signaling, how the coiled-coil/B-box framework drives self-association and repair complex formation, and how the PRY-SPRY domain contributes to partner recognition.

### 2.1. RING Domain: Ubiquitin E3 Ligase Activity

The N-terminus of MG53 contains a RING (Really Interesting New Gene) domain, a cysteine-rich zinc-binding motif that confers ubiquitin E3 ligase enzymatic activity [62,64,65]. This activity enables MG53 to catalyze the transfer of ubiquitin from E2 conjugating enzymes to selected substrate proteins, thereby regulating their stability, abundance, localization, and signaling output [66,67]. Although MG53’s membrane repair function does not strictly require its E3 ligase activity, this domain provides MG53 with a dynamic biochemical interface that links redox cues, metabolic state, and protein turnover to tissue integrity.

Several substrates have been reported for MG53 E3 ligase activity, with consequences that are often cell type- and context-dependent. Nutrient overload or high-glucose conditions have been reported to suppress AMP-activated protein kinase (AMPK) signaling through an MG53-dependent ubiquitin-proteasome mechanism. In this model, high glucose increases reactive oxygen species (ROS) and activates protein kinase B (AKT), which phosphorylates AMPK; this phosphorylation event facilitates MG53 recruitment to AMPKα and promotes MG53 E3 ligase-dependent ubiquitination, leading to AMPKα degradation and reduced AMPK activity in skeletal muscle [68]. During skeletal myogenesis, MG53 forms a complex with focal adhesion kinase (FAK) and the E2 enzyme ubiquitin-conjugating enzyme E2 H (UBE2H), and promotes ubiquitin-dependent, proteasome-mediated degradation of FAK [69]. Importantly, RING-disrupted MG53 mutants (e.g., C14A or ΔR) fail to induce FAK ubiquitination/degradation, establishing a requirement for MG53’s E3 activity during myogenic remodeling [69]. In the heart, MG53’s E3 ligase activity has been linked to protection in distinct injury contexts: in septic cardiac dysfunction, MG53 ubiquitinates activating transcription factor 2 (ATF2), thereby suppressing Toll-like receptor 4 (TLR4) transcription and limiting inflammation and cardiomyocyte apoptosis [50]; following cardiac ischemia/reperfusion (I/R), MG53 directly ubiquitinates receptor-interacting protein kinase 1 (RIPK1) to promote RIPK1 degradation and inhibit necroptosis [49].

Early studies agreed that MG53 can negatively regulate insulin signaling through ubiquitination of insulin receptor substrate-1 (IRS-1) [65,67], but differed on a key point: whether endogenous MG53 is consistently upregulated in metabolic disease and thereby acts as a causal driver of insulin resistance. Song and colleagues proposed a diabetogenic model in which MG53 is elevated in insulin-resistant states and contributes to metabolic dysfunction [65]. By contrast, Yi et al. showed that MG53 deficiency protects mice from high-fat/high-sucrose diet-induced insulin resistance through preservation of IRS-1, while also reporting that MG53 expression remains unchanged in human diabetic muscle and in several insulin-resistant mouse models [67]. Subsequent studies found no consistent increase in MG53 expression in diabetic or insulin-resistant muscle, including human samples and multiple rodent models, raising the possibility that differences in model systems, tissue sources, and protein detection methods may have contributed to the divergent conclusions [15,22,23,70]. Studies using validated, highly sensitive MG53 detection approaches suggested that circulating MG53 may decrease, rather than increase, in metabolic syndrome or diabetes, and that metabolic stress may alter MG53 trafficking or subcellular partitioning [23,70]. In parallel, sustained elevation of circulating MG53 in the tPA-MG53 transgenic mouse model increased regenerative capacity without impairing glucose handling or insulin signaling [22]. Consistently, a human case–control study found no association between circulating MG53 levels and diabetes status or key metabolic parameters [71]. Rather than serving as a primary and universal driver of insulin resistance, MG53 may exert context-, compartment-, and species-dependent effects, with its E3 ligase activity contributing to adaptive protein quality control responses under metabolic stress.

### 2.2. Coiled-Coil and B-Box Domains: Oligomerization and Repair Complex Formation

A defining feature of MG53 biology is its rapid, damage-triggered reorganization into higher-order assemblies that function as a nucleation platform at injury sites [18,20] (Figure 1B). This is driven primarily by coiled-coil-mediated self-association, with B-box dimerization further facilitating efficient higher-order assembly [18,63,72]. Importantly, this assembly is redox-dependent: MG53 monomers dimerize and form higher-order oligomers upon sensing oxidative changes in the local intracellular environment, precisely the conditions that occur when the plasma membrane is injured and cytosolic reducing potential is perturbed.

Mechanistically, oxidized milieus promote MG53 oligomerization through intermolecular disulfide bonding, with Cys242 acting as a critical redox sensor; mutation of C242 to alanine (C242A) disrupts oxidation-driven oligomerization and impairs repair capacity [18,72]. Leucine-zipper motifs within the coiled-coil region mediate MG53 intermolecular interactions and stabilize MG53 dimerization/oligomerization during repair, which is essential for MG53-mediated vesicle translocation toward the membrane injury site [72]. The coiled-coil domain is highly flexible and may help the repair scaffold adapt to the local membrane topology during injury [73]. B-box dimerization greatly enhanced C242-C242 disulfide bond formation under oxidative conditions and further facilitates efficient higher-order assembly [63]. Notably, Zn^2+^ influx and Zn^2+^ binding to Zn-finger motifs within the MG53 RING/B-box regions are required for MG53 vesicle recruitment and repair patch formation, suggesting that Zn^2+^ influx enables or potentiates MG53 assembly into a functional repair complex [74].

Oligomerized MG53 acts as a nucleation hub that recruits intracellular vesicles to membrane injury sites. Through its self-association and multivalent interactions, MG53 rapidly forms a scaffold that organizes a multi-protein repair complex at the membrane injury sites, key partners including dysferlin [75], caveolin-3 (CAV3) [75], polymerase I and transcript release factor (PTRF) [76], and annexins [77,78]. Importantly, MG53 oligomerization must be tightly regulated; excessive or mislocalized oligomerization could lead to aberrant protein aggregation. MG53’s sensitivity to the oxidative microenvironment provides a built-in regulatory mechanism, ensuring that oligomer formation occurs only when and where membrane damage has perturbed redox balance.

This redox-gated mode of assembly is a notable feature of MG53 and is well suited to the rapid, context-specific responsiveness required for membrane repair. Nevertheless, several structure-function relationships within this process, including how domain dynamics and partner recruitment are coordinated in vivo, remain incompletely defined and warrant further investigation.

### 2.3. PRY-SPRY Domain: Target Recognition and Membrane Localization

At the C-terminus, MG53 contains a PRY-SPRY domain, a hallmark interaction module in many TRIM proteins that confers target specificity. Structural analyses indicate that MG53 adopts an overall architecture distinct from TRIM20 and TRIM25 [61], underscoring how variation in PRY-SPRY positioning and dynamics may tune partner engagement across the TRIM family.

The central role of the MG53 PRY-SPRY region is to direct MG53 to damaged membrane surfaces enriched in exposed anionic phospholipids. Consistent with this, structural and biochemical studies show that MG53 can interact with phosphatidylserine (PS), a lipid normally restricted to the inner leaflet of the plasma membrane but rapidly externalized during cell injury, providing a plausible molecular basis for selective accumulation on perturbed membranes [63]. This phospholipid-binding capacity is important not only for intracellular repair but also for MG53’s activity as a circulating repair factor, enabling extracellular rhMG53 or serum-derived MG53 to bind directly to damaged cells and initiate repair processes from the outside.

Beyond lipid addressing, the PRY-SPRY domain is widely viewed as a major protein–protein interaction interface. In cardiomyocytes, MG53 has been proposed to scaffold a pro-survival signaling complex by bridging caveolin-3 and PI3K, with its N-terminal region binding caveolin-3 and its PRY-SPRY domain binding PI3K-p85, thereby promoting PI3K-dependent cardioprotective signaling (RISK) [79]. Another well-mapped target is calcium release-activated calcium channel protein 1 (Orai1), a key component of store-operated calcium entry (SOCE): MG53 binds Orai1 via its PRY-SPRY region and modulates extracellular Ca^2+^ entry in skeletal muscle, expanding PRY-SPRY function into calcium signaling regulation [80].

Overall, the PRY-SPRY domain endows MG53 with the specificity required to anchor the repair complex at precisely the injured membrane location. This domain represents the “addressing system” of MG53, ensuring that MG53’s redox-triggered oligomerization and scaffolding functions are deployed accurately and efficiently. A deeper understanding of PRY-SPRY interaction rules across lipids and protein targets will be essential for dissecting MG53’s pleiotropic biology and for engineering MG53-based therapeutics with improved precision.

## 3. Cryo-EM/ET Insights into MG53 Assembly and Membrane Targeting

Recent advances in cryo-electron microscopy (cryo-EM) and cryo-electron tomography (cryo-ET) have provided unprecedented insights into the higher-order structural organization of MG53, offering visualization of oligomeric architectures relevant to membrane repair. Prior to these structural studies, much of our understanding of MG53 assembly was inferred from biochemical assays, crosslinking experiments, and homology models derived from related TRIM family proteins. Cryo-EM/ET has now validated and expanded these models, revealing how MG53’s individual domains cooperate to form a dynamic, mechanically responsive repair scaffold [61,62,63].

A key advance is the demonstration that MG53 forms a homodimeric architecture with a relatively stable central “body” and more dynamic peripheral “wings” [61]. Complementing this, structural analysis identified an important role for the B-box-coiled-coil-SPRY region in redox-dependent oligomerization [63]. In particular, dimerization of the B-box could facilitate the formation of linear MG53 oligomers and promote C242-C242 disulfide bond formation under oxidative conditions [63]. Moreover, C242 is positioned within the coiled-coil region and is partially shielded by the B-box, while molecular dynamics simulations support a model in which conformational rearrangements within the B-box relative to the coiled-coil domain increase solvent exposure of C242 redox sensor, suggesting the dynamics of the B-box could regulate C242 disulfide bond formation and help expose C242 when necessary [63].

Another key study captured MG53 on lipid bilayers and developed a model for how membrane engagement can organize and activate the RING E3 ligase [62]. The RING/B-box modules are positioned toward the periphery of the assembly, consistent with their accessibility for partner engagement. Moreover, this model demonstrated that interaction between MG53 and PS-enriched membranes is necessary for MG53’s oligomeric assembly and is coupled to RING E3 activation, suggesting that MG53 can coordinate membrane-directed scaffolding with context-dependent ubiquitin signaling rather than operating in strictly separable “repair” versus “signaling” states [62].

These structural models also provide insights into lesion targeting. The PRY-SPRY domain is positioned at the distal end of the coiled-coil “arms”, where its apparent mobility could support partner engagement and membrane targeting [61,62,63]. Evidence also indicates that MG53 can interact with negatively charged lipids (particularly PS) [62], providing a basis for lesion-selective recognition of damaged membranes.

At the cellular scale, super-resolution fluorescence imaging provides complementary evidence for highly organized MG53 repair assemblies at lesions. 3D structured illumination microscopy (3D-SIM) showed a physiological Ca^2+^-dependent, interdigitating dysferlin-MG53 lattice concentrated at lipid-exposed lesion rims [81]. Over time, these concentrated lattices can expand into patch-like membrane structures during membrane resealing [81]. Together with cryo-EM/ET-derived models, these observations support the view that MG53 may function as a structural effector in addition to its signaling-related roles, and that its self-assembly into higher-order architectures may contribute to rapid and adaptable repair behavior by enabling repair scaffolds to accommodate diverse lesion sizes and membrane topologies.

Collectively, the insights gained from cryo-EM/ET reinforce the view of MG53 as a structurally dynamic, stress-responsive repair protein whose domain architecture and oligomeric transitions are well suited for rapid membrane sealing. Although whether and how these structural states directly govern extracellular MG53 function at damage-exposed membranes remains to be directly tested, they provide a valuable framework for future efforts in MG53 variant design and biomimetic engineering.

## 4. Regulation of MG53 Expression and Secretion

**Figure 2:**
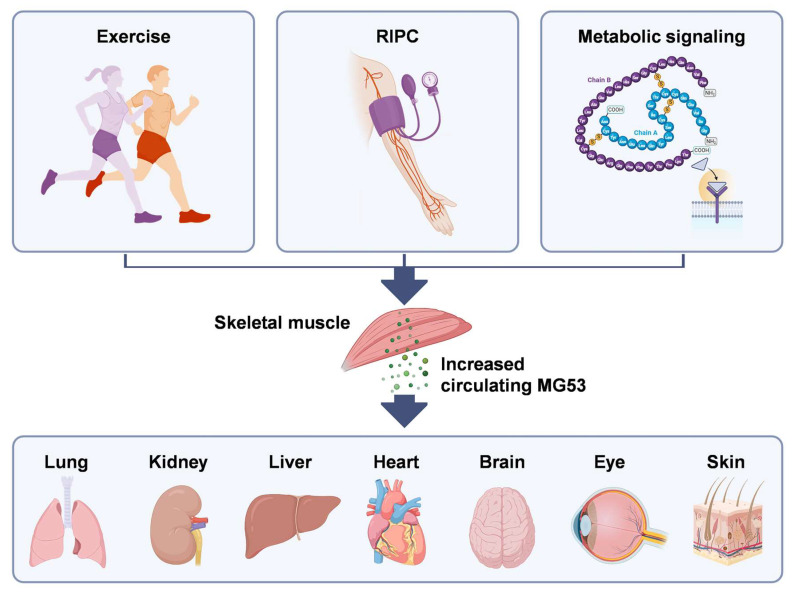
MG53 functions as a systemic regenerative myokine. MG53 is highly enriched in skeletal muscle, and its release into the circulation can be stimulated by exercise, remote ischemic preconditioning (RIPC), and metabolic cues such as insulin. Basal MG53 expression is low in most non-skeletal muscle tissues but can increase under stress, suggesting engagement of MG53-mediated protective pathways during pathological conditions. In many cases, such increases reflect recruitment of circulating MG53 released from skeletal muscle rather than substantial local induction within injured tissues. Created with BioRender.com.

MG53 expression is highly enriched in striated muscle, reflecting its specialization as a guardian of myofiber integrity. Striated muscles maintain substantial intracellular reserves of MG53, poised for rapid deployment upon membrane injury. Basal MG53 expression in most other tissues is low but can increase under stress, suggesting that non-muscle cells may engage MG53-mediated protective programs during pathological states [16,35,48,82] (Figure 2). Current evidence suggests that such an increase in MG53 may not primarily reflect local upregulation of MG53 expression within the injured tissue, but rather recruitment of circulating MG53 released from skeletal muscle and delivered to sites of tissue damage.

### 4.1. Regulation by Exercise and Physiologic Activity

Exercise is one of the most potent physiological stimuli for MG53 mobilization into the circulation. Muscle contraction imposes mechanical strain on the sarcolemma, producing repetitive microtears accompanied by local perturbations in ionic gradients and redox balance. MG53 responds rapidly to these perturbations by translocating to sites of injury, where it orchestrates membrane repair [20]. At the systemic level, acute or exhaustive exercise increases circulating MG53 levels, reflecting mobilization of MG53 from contracting myofibers into the bloodstream [36,83]. Notably, prolonged exercise training does not increase MG53 abundance within muscle, indicating that exercise-associated elevations in circulating MG53 mainly arise from myocyte release [83], potentially through a combination of regulated export and transient leakage from mechanically stressed fibers. Overall, this exercise-induced mobilization of MG53 supports a model in which MG53 functions as a stress-responsive circulating myokine that links muscle activity to systemic tissue resilience.

### 4.2. Remote Ischemic Preconditioning (RIPC)

An increasingly intriguing facet of MG53 biology concerns its response to systemic stress induced by RIPC. RIPC, typically induced by transient, repetitive I/R cycles in a limb, has long been recognized to confer protection to distant organs, particularly the heart, through integrated humoral and neural mechanisms, although the key circulating mediators remain poorly defined [84]. Building on the emerging concept that skeletal muscle can release protective myokines in response to transient ischemic stress, RIPC has been proposed as a physiologically tractable strategy to mobilize endogenous, muscle-derived cytoprotective factors.

Consistent with this idea, RIPC has been reported to induce a rapid rise in circulating MG53, as assessed by plasma immunoblotting following RIPC [85]. Another study in cardiac ischemic preconditioning demonstrates that preconditioning/oxidative signaling can promote MG53 secretion in mice, supporting the link between preconditioning stimuli and extracellular MG53 availability [86]. These findings together raise the testable hypothesis that RIPC-evoked MG53 release may contribute to enhanced membrane repair capacity in remote organs (e.g., heart, kidney, and lung) under stress. The ability of skeletal muscle to acutely release MG53 in response to controlled ischemic stress positions RIPC as a noninvasive, autologous strategy for enhancing endogenous MG53 bioavailability. This raises potential translational implications, as RIPC could be harnessed perioperatively or in critical care settings to elevate circulating MG53 and improve tissue resilience during ischemia, reperfusion, or inflammatory injury. Although the magnitude and kinetics of RIPC-induced MG53 secretion in humans remain to be fully defined, these early findings highlight a promising avenue for exploiting physiological stress responses to augment systemic membrane repair capacity.

### 4.3. Influence of Metabolic State and Aging

MG53 expression and secretion are also influenced by metabolic conditions. Current literature supports a context-dependent model. Early work reported elevated MG53 in mouse models of insulin resistance [65], whereas multiple independent studies found no consistent upregulation of MG53 in diabetic or insulin-resistant settings, including analyses of human diabetic samples and several animal models [15,22,28,67,70,71]. Beyond expression, insulin stimulation has been shown to trigger MG53 release into the circulation [23]. Together, these findings argue against MG53 expression as a primary causative driver of metabolic disease and instead support the possibility that metabolic stress alters MG53 trafficking (e.g., mitochondrial sequestration) as part of an adaptive protective response. Importantly, long-term elevation of circulating MG53 in the tPA-MG53 mouse model enhanced tissue regenerative capacity without compromising glucose handling or insulin signaling, supporting the view that extracellular MG53 can be engaged therapeutically without necessarily perturbing systemic metabolic homeostasis [22].

Aging adds another regulatory layer relevant to tissue resilience. Available data suggest that MG53 abundance can change with age. In *mdx* mouse model of Duchenne muscular dystrophy, MG53 protein levels were reduced in aged extensor digitorum longus (EDL) muscle [87]. In the heart, MG53 levels are also reduced in aged myocardium, and MG53 has been reported to suppress nuclear factor kappa B (NF-κB) activation to mitigate age-related heart failure, supporting the concept that age-associated MG53 insufficiency may contribute to diminished cardiac stress tolerance [56]. Consistent with the essential role of MG53 in long-term membrane integrity, MG53-deficient mice develop age-dependent myopathy in their skeletal muscles and show increased vulnerability to cardiac I/R injury [57,58]. Whether circulating MG53 changes with healthy aging, and how this relates to declining repair capacity, remains unresolved and will require longitudinal and multi-omics profiling. Nonetheless, age-related reductions in MG53 may contribute to the diminished resilience of aged tissues, making restoration of MG53 activity a potential strategy to help counteract age-associated degeneration.

### 4.4. Mechanisms of MG53 Secretion

MG53 lacks a classical signal peptide, and its presence in the extracellular space therefore implies non-canonical release mechanisms. What remains unresolved is how MG53 crosses the membrane. Current data do not yet establish whether extracellular MG53 is released predominantly through (i) stress-coupled membrane “leakage” from injured fibers, (ii) regulated export linked to vesicle trafficking/exocytosis that accompanies repair, or (iii) packaging into extracellular vesicles such as microvesicles or exosomes. These pathways are likely activated under conditions of membrane stress, oxidative disruption, or mitochondrial dysfunction. Defining the molecular routes, kinetics, and carrier forms of MG53 release across physiological versus pathological contexts remains an active area of investigation with significant therapeutic implications, particularly for enhancing systemic MG53 bioavailability.

## 5. The Unique Role of MG53 as a Myokine for Human Cardioprotection

MG53 was first established as an intracellular organizer of acute membrane repair in striated muscle, but subsequent work demonstrated that MG53 is also present in the circulation and can function as a systemic regenerative factor [16,35,48]. The recognition of MG53 as a myokine significantly broadened the conceptual landscape of membrane repair biology. This endocrine dimension adds a profound new layer to our understanding of muscle-organ communication, with particularly direct relevance to the heart.

### 5.1. Human Vulnerability and the Importance of Circulating MG53

**Figure 3:**
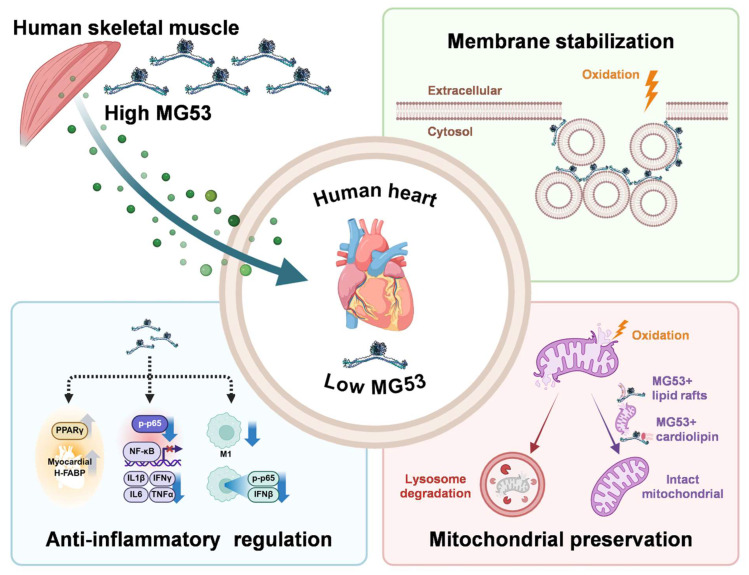
Skeletal muscle-derived MG53 functions as an endocrine repair factor for the human heart. Human cardiac tissue expresses minimal intrinsic MG53 and may therefore rely more heavily on circulating MG53, produced predominantly by skeletal muscle, to augment repair capacity during injury. During cardiac stress, MG53 is proposed to support cardiomyocyte survival through membrane stabilization, mitochondrial preservation, and attenuation of inflammatory signaling. Created with BioRender.com.

A key biological insight with profound translational implications is that adult human cardiac tissue expresses minimal intrinsic MG53, especially when compared to rodent myocardium or human skeletal muscle [39,56,60]. This species- and tissue-specific deficit suggests that the human heart may depend more heavily on circulating MG53, secreted primarily by skeletal muscle, to supply repair capacity during injury (Figure 3).

### 5.2. Circulating MG53 as an Endocrine Repair System for the Heart

Heart failure (HF) following myocardial infarction (MI) remains a persistent clinical challenge. Despite major advances in reperfusion, antiplatelet therapy, and neurohormonal blockade, current standard-of-care interventions do not directly augment the heart’s immediate capacity to stabilize injured cardiomyocytes and repair membrane damage during I/R stress [88,89]. In this context, multiple preclinical studies demonstrate that exogenous MG53 reduces infarct size and preserves ventricular function, including in a large-animal (porcine) model [28,56,57], providing a direct translational bridge between circulating MG53 availability and cardioprotection.

The recognition that MG53 can act as a myokine redefines its protective potential in human cardiology. Current evidence supports a model in which circulating MG53 can engage injured cardiomyocytes by binding to exposed PS and other damage-associated lipids. Once recruited to damaged membranes, MG53 promotes membrane resealing and preserves cardiomyocyte integrity, thereby extending the MG53-dependent repair program beyond the cell of origin. This endocrine extension of the membrane repair machinery provides an important compensatory mechanism for the human heart, where endogenous MG53 expression appears insufficient to support a robust autonomous repair response. Collectively, accumulating findings support an endocrine “repair reservoir” model in which skeletal muscle-derived circulating MG53 can contribute to cardiac repair, although more direct validation and deeper mechanistic investigation are still needed.

### 5.3. Mechanisms of MG53-Mediated Cardioprotection

During cardiac I/R, cardiomyocytes experience intense membrane fragility, calcium overload, ROS bursts, and mitochondrial dysfunction [90,91]. Beyond acute mechanical repair, MG53 supports cardiomyocyte survival through complementary mechanisms including mitochondrial stabilization and anti-inflammatory regulation (Figure 3). Among these, membrane stabilization is the most direct and best-established axis, whereas mitochondrial preservation and inflammatory modulation are currently supported by a smaller and more context-dependent body of evidence.

#### 5.3.1. Membrane Repair

MG53 counteracts the earliest stage of cardiomyocyte injury during I/R: sarcolemmal microdisruptions that precipitate pathological Ca^2+^ influx and amplify downstream death pathways. Upon membrane damage, MG53 selectively accumulates on damage-exposed membrane disruptions within seconds and promotes efficient membrane resealing, thereby helping preserve ionic homeostasis and limiting injury propagation [18]. The physiological relevance is also supported by the observation that autoantibodies targeting MG53 compromise membrane repair and are associated with inflammatory myopathy [19]. Beyond acute sarcolemmal resealing, MG53 also preserves the integrity of the cardiac T-tubule network under pathological stress. In a pressure-overload model, MG53 deficiency did not affect T-tubule development or baseline maintenance, but markedly worsened stress-induced T-tubule disruption, Ca^2+^ handling abnormalities, hypertrophy, and cardiac dysfunction, identifying MG53 as a stress-inducible safeguard of membrane microarchitecture and excitation-contraction coupling [92].

#### 5.3.2. Mitochondrial Stabilization

Beyond plasma membrane resealing, MG53 can preserve mitochondrial integrity under I/R stress. Endogenous MG53 has been reported to localize to mitochondria in skeletal muscles derived from high-fat diet-fed mice [70] and to be enriched in the mitochondria fraction of mouse hearts subjected to ischemic preconditioning [93]. Moreover, MG53 has been shown to translocate to mitochondria after myocardial infarction injury, bind the mitochondrial lipid cardiolipin, reduce superoxide generation, and restrain injury-associated mitophagy, collectively supporting mitochondrial preservation as a second protective axis that complements membrane stabilization [17]. This mitochondria-directed action provides a model in which MG53 dampens the ROS-mitochondrial dysfunction feedback loop that otherwise drives cardiomyocyte death and adverse remodeling during reperfusion. Whether this effect is mechanistically separable from MG53’s membrane-repair function, or instead represents a downstream extension of membrane stabilization, remains unclear. It will also be important to determine whether MG53 interacts with additional mitochondrial regulatory proteins or influences stress-responsive transcriptional programs linked to mitochondrial quality control.

#### 5.3.3. Modulation of Inflammatory and Immune Responses

Membrane injury and mitochondrial dysfunction can propagate post-ischemic inflammation through the release of damage-associated molecular patterns (DAMPs) and induction of cytokine programs. By stabilizing injured membranes and preserving cellular integrity, MG53 may therefore help limit DAMP-driven inflammatory amplification. In failing human hearts and aged mouse hearts, MG53 levels are reduced and correlate with elevated NF-κB activity; notably, longitudinal rhMG53 treatment mitigated cardiac dysfunction in aged mice, with evidence linking benefit to suppression of NF-κB-mediated inflammatory signaling [56]. In a cecal ligation and puncture (CLP) model, MG53 supplementation has also been reported to protect against sepsis-induced myocardial dysfunction by upregulating peroxisome proliferator-activated receptor alpha (PPARα)-mediated anti-inflammatory signaling and reducing release of the cardiac injury marker heart-type fatty acid-binding protein (H-FABP) [94]. In macrophages, MG53 knockdown increases interferon beta (IFNβ) production and enhances NF-κB signaling, consistent with heightened M1-like inflammatory programs in the absence of MG53 and suggesting that MG53 can dampen pro-inflammatory macrophage states [32]. Importantly, these anti-inflammatory effects cannot be explained solely as a secondary consequence of membrane stabilization. Future studies are warranted to investigate the possibility that MG53 participates in inflammatory signaling networks through transcriptional regulations.

Together, these findings support an immunomodulatory arm of MG53 cardioprotection that reduces secondary inflammatory damage and improves the tissue environment for functional recovery. More broadly, MG53’s dual role as a structural defender and a regulator of the inflammatory landscape is particularly important in conditions such as acute respiratory distress syndrome (ARDS), sepsis, and I/R injury, where membrane disruption and immune dysregulation occur simultaneously.

## 6. Therapeutic Development and Clinical Translation

Given the minimal endogenous MG53 expression in the adult human heart, therapeutic augmentation of MG53 activity represents a plausible strategy to compensate for this intrinsic vulnerability and enhance cardiomyocyte resilience. Beyond acute MI, MG53-based approaches may also be relevant to other cardiac settings in which membrane fragility and/or mitochondrial dysfunction contribute to injury, including anthracycline-induced cardiotoxicity, heart failure with preserved ejection fraction (HFpEF), and perioperative cardiac injury, although disease-specific validation remains needed.

Unlike many pharmacologic agents that modulate intracellular signaling or systemic hemodynamics, MG53 directly targets an upstream biophysical determinant of injury: loss of plasma membrane integrity. This approach offers several conceptual advantages. First, it is broadly applicable across diverse triggers of membrane stress (e.g., I/R, mechanical stress, metabolic overload, toxins). Second, it operates rapidly, within the therapeutic window of reperfusion. Third, by stabilizing injured cells, it may create a foundation for downstream cytoprotective and reparative programs to operate more effectively.

### 6.1. Recombinant Human MG53

Recombinant human MG53 (rhMG53) is currently the most extensively studied MG53-based therapeutic modality and has demonstrated efficacy across a broad spectrum of preclinical disease models. Following systemic or local administration, rhMG53 binds to exposed phospholipids on injured cells and helps preserve cellular integrity. rhMG53 has been reported to reduce tissue damage and improve functional outcomes in preclinical models of myocardial infarction [28,56], lung injury [24,25,34,41,55], acute kidney injury [26,43,82], skeletal muscle injury/degeneration [16,20,21,37,45], brain injury [29,95], and neurodegeneration [96].

Pharmacokinetic measurements indicate that rhMG53 is detectable in plasma within a therapeutically relevant window, although its native circulating half-life is relatively short and may benefit from half-life extension strategies [64]. The efficacy observed after multiple administration routes (e.g., intravenous, intramuscular, intratracheal) supports the feasibility of systemic and local delivery of rhMG53 across organ systems. Overall, available data are consistent with an encouraging preclinical safety profile of rhMG53 in rodents and large-animal studies, without clear evidence of toxicity reported to date [15,22,28]. However, additional work will be needed to define optimal dosing schedules, target engagement biomarkers, durability of benefit, and anti-drug antibody (ADA) risk under chronic exposure.

### 6.2. Gene Therapy

Gene therapy offers a potential strategy for sustained augmentation of MG53 activity. Adeno-associated virus (AAV)-mediated expression of MG53 in skeletal muscle is conceptually attractive because muscle serves as the body’s natural reservoir of MG53 and may provide a durable source of circulating protein. In this framework, skeletal muscle could function as a long-term supply compartment for systemic repair support, particularly for tissues with limited intrinsic MG53 expression.

Preclinical studies have reported the benefit of AAV-mediated MG53 expression in muscular dystrophy and heart failure [44,97], inflammatory bowel disease [52], and neuropathic pain [98]. The development of muscle-tropic AAV capsids, such as MyoAAV [99], can enhance transduction efficiency and reduce off-target expression, allowing for lower vector doses and improved safety margins. Dual-promoter expression strategies enabling combined skeletal and cardiac expression may further expand this approach. For human cardioprotection, gene therapy could provide a long-term supplementation strategy [100], particularly for patient populations with diminished skeletal muscle mass, such as the elderly or those with cachexia, in whom endogenous muscle-derived MG53 availability may already be limited. Nevertheless, translation will require further validation of MG53-based constructs in disease-related models, as well as careful attention to dose control, tissue specificity, expression durability, and long-term safety.

### 6.3. Biomaterial and Hydrogel Delivery Systems

Localized delivery through biomaterials provides a complementary strategy to systemic administration [101,102], enabling site-specific enrichment and sustained release of bioactive MG53 in injury microenvironments. Incorporating MG53 into hydrogels, tissue scaffolds, or surgical sealants can enable sustained, site-specific release of bioactive protein, thereby concentrating its effects in regions where membrane instability and metabolic stress are most pronounced. By reducing reliance on systemic distribution, biomaterial-based delivery provides a targeted method to exploit MG53’s membrane-stabilizing and cytoprotective properties in settings where localized tissue vulnerability drives clinical outcomes.

In the context of engineered delivery, MG53-based formulations have been developed to enhance stability and extend exposure. For example, an MG53-mediated biomimetic nanotherapeutic has been reported to enable sustained delivery and extend the half-life of rhMG53 proteins in circulation, with superior efficacy in protecting injured cardiomyocytes and preserving heart function post-myocardial infarction [103]. In dermal repair, thermoresponsive hydrogels have been used to localize and sustain rhMG53 at wound sites, accelerating closure and reducing scar formation [27]. In diabetic wounds, a ROS-scavenging thermosensitive gel enabling sustained rhMG53 release improved chronic wound healing in db/db mice and supported regenerative features such as hair follicle development [104]. Hydrogel-based MG53 delivery has also been extended to other epithelial injuries, including corneal wound healing [105], underscoring the broader utility of localized, controlled MG53 delivery.

More broadly, integrating MG53 into regenerative constructs is an attractive strategy to enhance cell survival in harsh injury microenvironments. However, it still requires dedicated investigation to develop an MG53-based myocardial “patch” that can support the infarct border zone, reduce wall stress, and mitigate maladaptive ventricular remodeling. Similarly, incorporating MG53 into regenerative skin grafts or engineered tissues may offer a route to improve graft integration and vascularization, although these applications remain largely conceptual and await further validation.

### 6.4. Safety Considerations and Key Translational Bottlenecks

Safety evaluation is essential for therapeutics targeting fundamental cellular processes such as membrane repair. Despite early concerns raised by selected rodent models, a growing body of pharmacologic evidence from rodents or large animals supports an encouraging preclinical safety profile for rhMG53 administration [15,22,28,67,70]. Reports to date also suggest low immunogenicity risk, with repeated dosing of rhMG53 in dogs showing unchanged pharmacokinetics, a pattern consistent with limited ADA liability [26,56].

Taken together, these findings support continued IND-enabling development of MG53-based approaches. At the same time, important translational bottlenecks remain. Some reflect unresolved MG53 biology, such as incomplete mechanistic understanding of MG53 secretion and trafficking, extracellular carrier forms, and tissue selectivity. Others are practical development challenges, including the relatively short native half-life of E.coli-derived rhMG53, uncertainty regarding optimal dosing frequency and formulation format, limited biomarkers of target engagement in vivo, and the need to define realistic first-in-human indications most likely to benefit from MG53-based therapy. Chronic ADA liability under prolonged exposure will also require continued evaluation.

## 7. Conclusions and Future Directions

MG53 has emerged as a multifunctional myokine with roles that extend well beyond its original characterization as a skeletal muscle membrane repair protein. Accumulating evidence supports the view that MG53 can function as a systemic regenerative factor whose extracellular actions contribute to tissue protection across multiple organ systems. This concept may be particularly important for the human heart, where intrinsic MG53 expression appears to be very limited relative to skeletal muscle and rodent myocardium.

Despite recent progress, several important questions remain unresolved. One major gap concerns the mechanisms regulating MG53 secretion. Unlike classical secreted proteins, MG53 appears to utilize non-canonical export pathways linked to redox perturbations, mechanical stress, and mitochondrial signaling. It will be important to determine whether stress-responsive mitochondria-linked pathways, including mitochondrial-derived peptides, can modulate MG53 secretion and thereby influence systemic resilience. Dissecting this network will be essential for understanding how MG53 secretion might be therapeutically modulated in humans, especially in aging or sarcopenic populations with reduced myokine reserve.

A related uncertainty concerns MG53 receptors or accessory proteins. Although binding to exposed phosphatidylserine provides a plausible explanation for lesion-selective membrane engagement, this mechanism alone may not fully explain the breadth or tissue specificity of MG53 action. Whether additional receptors, co-factors, or membrane-associated partners contribute to MG53 internalization, signaling, or organ selectivity remains an open question. Resolving these mechanisms would significantly strengthen the conceptual framework of extracellular MG53 biology and could inform the design of improved therapeutic formats with greater tissue precision.

The intersection of MG53 biology with immune modulation and NETosis also warrants further investigation. Membrane disruption and uncontrolled inflammation often co-occur in diseases such as ARDS, pneumonia, trauma-induced lung injury, and sepsis. In this setting, MG53-based membrane stabilization and NETosis targeting approaches (such as anti-CitH3 monoclonal antibodies) may offer mechanistically complementary effects. In the acute phase, such a combination could potentially provide dual benefit by rapidly limiting membrane injury and dampening DAMP-driven inflammatory amplification. Over a longer time frame, this strategy may also help reduce persistent immune dysregulation and improve the tissue microenvironment for repair. Although this concept remains hypothetical, it provides a plausible framework for simultaneously targeting the structural and immune-mediated components of tissue injury.

On the translational side, the manufacturing of MG53-based therapeutics remains a key challenge and opportunity. Most published preclinical studies have utilized rhMG53 derived from prokaryotic expression systems such as E. coli, which enables scalable production but may not fully preserve structural features relevant to redox-dependent oligomerization and proper folding. Mammalian expression platforms, such as CHO-based systems, offer the potential to generate MG53 proteins with improved biochemical fidelity and therapeutic performance. However, achieving commercially viable titers in such systems will require substantial advances in vector engineering, promoter design, culture optimization, and downstream purification. Comparison of product quality and bioactivity across expression systems is also an important step in advancing MG53-based therapeutics.

Overall, MG53 represents a promising but still mechanistically incomplete translational candidate, particularly in cardiac settings. Its membrane-directed mode of action distinguishes it from conventional signaling-targeted agents and may offer advantages in diseases characterized by acute or chronic membrane fragility. At the same time, further progress will depend on resolving key questions surrounding secretion biology, extracellular carrier forms, tissue selectivity, pharmacokinetics, and long-term safety. Continued investigation across these areas will help clarify where MG53-based interventions are most likely to succeed and determine whether this distinctive repair factor can be effectively translated into clinical therapy.

## Figures and Tables

**Table 1:** Current evidence landscape of key concepts in MG53 biology.

Category	Well Supported	Supported but Incomplete *	Open Questions
Circulating MG53 exists and is protective in disease/injury models	√		
Adult human heart has low intrinsic MG53	√		
Human heart dependence on circulating MG53		√	
Membrane repair	√		
Mitochondrial protection		√	
Anti-inflammatory/immunomodulatory effects		√	
Extracellular lesion targeting via damage-exposed lipids		√	
Tissue selectivity mechanisms			√
Secretion route, extracellular carrier form, and receptor/accessory proteins			√
Readiness for clinical translation		√	

* Further investigation across datasets and disease contexts, together with deeper mechanistic studies, is warranted.

## Data Availability

No new data were created or analyzed in this study. Data sharing is not applicable to this article.
